# The Role of the Crosstalk Between Gut Microbiota and Immune Cells in the Pathogenesis and Treatment of Multiple Myeloma

**DOI:** 10.3389/fimmu.2022.853540

**Published:** 2022-03-31

**Authors:** Marcin Jasiński, Jarosław Biliński, Grzegorz W. Basak

**Affiliations:** ^1^ Department of Hematology, Transplantation and Internal Medicine, Medical University of Warsaw, Warsaw, Poland; ^2^ Doctoral School, Medical University of Warsaw, Warsaw, Poland; ^3^ Human Biome Institute, Gdańsk, Poland

**Keywords:** multiple myeloma, gut microbiota, intestinal immune system, fecal microbiota transplantation, B cell, plasma cell

## Abstract

Around 10% of all hematologic malignancies are classified as multiple myeloma (MM), the second most common malignancy within that group. Although massive progress in developing of new drugs against MM has been made in recent years, MM is still an incurable disease, and every patient eventually has relapse refractory to any known treatment. That is why further and non-conventional research elucidating the role of new factors in MM pathogenesis is needed, facilitating discoveries of the new drugs. One of these factors is the gut microbiota, whose role in health and disease is still being explored. This review presents the continuous changes in the gut microbiota composition during our whole life with a particular focus on its impact on our immune system. Additionally, it mainly focuses on the chronic antigenic stimulation of B-cells as the leading mechanism responsible for MM promotion. The sophisticated interactions between microorganisms colonizing our gut, immune cells (dendritic cells, macrophages, neutrophils, T/B cells, plasma cells), and intestinal epithelial cells will be shown. That article summarizes the current knowledge about the initiation of MM cells, emphasizing the role of microorganisms in that process.

## 1 Introduction

Multiple myeloma (MM) is a hematological neoplasm deriving from clonal plasma cells. In almost every case, it is preceded by a premalignant stage called monoclonal gammopathy of undetermined significance (MGUS) (1, 2). In 3-4% of the whole population over the age of 50, the diagnosis of MGUS could be stated (3). The median age at the time of diagnosis of MM is approximately 70 years (4). The global incidence of MM steadily increases, which can be only partly explained by aging, with the highest score in Western European, North American, and Australasian populations reaching in 2016 about 5 cases per 100 000 persons. In 2019 the global incidence of MM amounted to 155 688 cases, compared to 138 509 in the year 2016. The age-standardized incidence rate (ASIR) was 1.92/100 000 in 2019. During the 2019 year, 113 474 deaths were noted due to MM, whereas 98 437 were in 2016. That short period of three years shows the dynamics of the new MM cases increase. From 1990 to 2016, the incidence of new MM cases increased by 126% (52.9% was attributed to aging, which is typical for cancers that mainly affect the older population), while deaths due to MM increased by 94% (5, 6). The incidence of MM in the population <30 years is infrequent (0.02-0.3%) (7). Fortunately, the prognosis for patients with MM significantly improved during the last years, which is due to many new drugs, better availability of autologous hematopoietic stem cell transplantation (ASCT), and constantly emerging new therapies such as CAR-T cells (8). To better illustrate the progress: the 5-year survival rate of MM in 1975-1977 was 25% and reached 49% in 2005-2011 (9).

As mentioned before, almost all cases of MM pass through an utterly asymptomatic phase referred to as MGUS, in which monoclonal, malignant in their nature plasma cells live in the patient’s body (2). Normal plasma cells carry on their surface the following combination of antigens: CD19^+^/CD56^-^/CD45^+^/CD38^+^, while the malignant plasma cells are losing CD19 and CD45 and acquiring CD56 (10). The threshold, when the abnormal plasma cells are still in a pre-cancerous entity, MGUS, is set on less than 10% of all bone marrow mononuclear cells (11). The oncogenesis is usually initiated within germinal centers of the lymph node during the isotype class switching and somatic hypermutation (SHM) occurrence (12). The leading role in the normal plasma cells transformation into malignant ones is attributed to cyclin D family proteins mutations enabling G1/S transition (13). Only 1-2% of MGUS patients progress to symptomatic MM per year (14). To become malignant, plasma cells must gain the proliferation and growth potential by self-renewing clone.

The two oncogenes believed to play a critical role in that process are Ras and Myc (15, 16). Interestingly, the mutations found in MM cells are also largely present at the MGUS stage, suggesting that genetic mutations are necessary but insufficient for myeloma development (17). The bone marrow environment plays a complementary role in that process. In addition to genetic factors and aging, environmental factors appear critical to forming a cancerous cell in MM. During our lifetime, our body cells, especially immunocompetent cells located in the lymphatic tissues of the structures that separate us from the outside world, e.g., in the intestines, skin, or liver, interact millions of times with various environmental factors - animate and inanimate. The more environmental signals for recombination and proliferation, the greater the likelihood of mutation in plasma cells, as in any other. It seems logical that chronic antigenic stimulation provokes many rounds of proliferation and selection of B cells, which means an increased risk of mutational changes starting oncogenesis when not repaired. Finally, the last stage of the disease is associated with stroma-independent growth and results in extramedullary diseases or plasma cell leukemia (PCL). The main pathway in this process is characterized by constitutive NF-κB activation, which influences the expression of adhesion molecules, such as VLA-4 (18).

In our previous work, we have described the role of the gut microbiome in pathogenesis, biology, and treatment of plasma cell dyscrasias (19). This review will gather all the information about the sophisticated interplay between the immune cells and the gut microbiota and how this could potentially lead to MM development.

## 2 Gut Microbiota – Significance During Our Life

One of the most surprising data regarding the first steps in gut colonization was that gut microbiota starts its development already *in utero.* Previously, the fetus’s intestine was considered germ-free, but that view was challenged with the results of a few studies. The microorganisms were detected in the amniotic fluid (20, 21), umbilical cord (22), placenta (23), and the most critical – meconium, which is the first excretion that derives from all that has been ingested or secreted before the delivery (24, 25). What is particularly interesting, in the mice model, microorganisms within the fetus’s gut resemble those which are colonizing the mother’s intestine (24). Therefore, these microbes should efflux the mother’s systemic circulation to reach the placenta.

Moreover, during the late pregnancy, the intestinal translocation of bacteria to the vessels is enhanced, which could play a role in the initial colonization of the fetus’s gut (26). A study conducted by Gosalbes et al. showed that the gut microbiota of infants during their first weeks of life includes the microorganisms found in the meconium, which were still detectable even seven months after birth (27). In addition, Brosseau et al. recently presented the study results, which shows that supplementation of prebiotics for pregnant women leads to the transmission of specific microorganisms and immune factors from mother to fetus allowing the development of the tolerogenic immune system imprinting that influences other health outcomes (28). However, these data contradict the recently published work, which shows that gut colonization starts after birth and bacteria found in meconium were the effect of skin contamination (29).

Right after birth, the gut is being rapidly colonized, and during that period, the mode of delivery plays a crucial role in establishing gut microbiota composition. For example, infants delivered vaginally possess the gut microbiota, mainly consisting of lactobacilli living in high abundance in the vagina (30). On the other hand, infants born through C-section are frequently colonized by the microorganisms such as *Clostridium* species and facultative anaerobes. Moreover, infants delivered by C-section are colonized by the *Bacteroides* genus with delay (31), and only 41% of their fecal microbiota is identical to the mother’s gut microbiota composition (72% in vaginally delivered infants) (32).

The gut microbiota composition during the first year of life changes, while the diversity of microorganisms colonizing the gut increases (33). Its composition resembles more and more of that seen in adults, but it takes another two years to establish a typical pattern of adult-like microbiota (34, 35). However, some studies showed that the maturation of human gut microbiota lasts for more than the first three years of life and can change its composition even till 12 years (36). The whole process of intestinal colonization by newer and newer microorganisms is remarkably similar to the dynamic development and growth of the repertoire of immunocompetent cells. These are mechanisms that go hand in hand, at the same time, and are strongly interdependent.

The impact of proper gut microbiota development is evident regarding the risk of immune disorders. Lack of balanced gut microbiota can result in various autoimmune and atopic diseases (37, 38). It is not surprising given the fact that the largest area of contact between microbes and immune cells is within the intestine. Our immune system is constantly stimulated by the enormous plethora of ligands presented by microorganisms colonizing the gut, such as lipopolysaccharides (LPS), flagellin, or unmethylated CpG motifs (39). These ligands shape the further differentiation of naïve T cells into T regulatory type (Treg) or the Th1, Th2, and Th17 cells (40). Tregs can inhibit the differentiation of naïve T cells towards Th types (41), suppress eosinophils, basophils, mast cells (42), and the production of immunoglobulin (Ig) E (43). Conversely, different types of Th cells can inhibit the other ones amplifying through that process the immune response (44).

For a long time, the researchers were focused on the role of balance between Th1 and Th2 cells. Excessive activation of one type of cell causes autoimmune and chronic inflammatory diseases (Th1) or allergic diseases (Th2) (45, 46). The role of Th17 cells in diseases classically associated with an imbalance of Th1/Th2 activation was also shown (47). Taking into consideration the role of balance between Treg and Th cells and also the fact that such balance is closely related to the composition of gut microbiota, it leads to the conclusion that gut microbiota is the initial factor in the pathogenesis of a wide variety of chronic inflammatory, allergic and autoimmune disease (48, 49).

There is also one other proof of how vital well-balanced gut microbiota is for maintaining the immune system in shape. Experiments on germ-free mice, free of any microorganisms, showed that gut microbiota is obligatory for Tregs differentiation (50). Other experiments showed that different bacteria and their products induce the activation of Tregs in mice (51). On the other hand, segmented filamentous bacteria (SFB) facilitate the differentiation of naïve T cells towards proinflammatory Th17 cells in mice (52). Together, these experiments showed us the key role of balanced gut microbiota in health and disease.

## 3 The Role of the B-Cell Chronic Stimulation in Multiple Myeloma Pathogenesis

The process of immunoglobulins (Ig) production starts within the germinal centers (GCs) of secondary lymphoid organs. This is where naïve B cells encounter T cells accountable for selecting B cells eligible for future combat against pathogens or antigens (53). Given that, one can easily conclude that the whole process of Ig production starts there – in the secondary lymphoid organs, especially in the gut-associated lymphoid tissue (GALT), and that is where the defense of the whole organism begins.

In the GCs, B cells are selected based on the higher affinity of B-cell receptors (BCR) towards the antigen. This is the initial step in immunity organization that will last for long years (53). During that process, the naïve B cells undergo two sophisticated DNA changes by which only B cells with the highest affinity against the antigen are selected. One of these processes is somatic hypermutation (SHM) with antigen selection, and the second one – immunoglobulin heavy chain (IgH) switch recombination. These two types of DNA modifications are the source of mutations and breaks of double-strand DNA, sometimes also in oncogenes (54). When the oncogene is positioned near the site of the Ig enhancer, then it results in dysregulation and potent proliferation of B cells. These are the initial cells that will constitute multiple myeloma (MM) (55). Many B cell neoplasms share the same feature, which are the translocations that are mediated by errors during recombination in V (variable), D (diversity), and J (joining) gene segments or the abovementioned two more subtle changes in DNA sequence (56).

The association between chronic intracellular infection with viruses [HCV, HSV, EBV (57)] or bacteria [*Helicobacter pylori* (58)] and the increased risk of neoplasms development was shown many years ago. It is now established that up to 20% of malignancies are microbiota-dependent (59). The transformation of a normal cell into malignant may occur indirectly *via* chronic antigenic stimulation of the BCR or directly *via* B cell infection and transformation (60). The main proof for the role of chronic antigenic stimulation in the pathogenesis of MGUS and MM is the specificity against some viruses of monoclonal Ig produced by mutated clone (61–63). Moreover, in some cases, the antiviral therapy against chronic HCV infection alone was sufficient to reach the regression in the MM that had features compatible with MGUS (64–66). On the other hand, patients with Gaucher’s disease have an increased risk of transforming normal B cells into myeloma cells (67). Some reports indicate that lyso-glucosylceramide 1 (LGL1) and lyso-phosphatidylcholine (LPC), which are accumulated in Gaucher’s disease, become antigens that drive the selection of B cells and therefore contribute to the pathogenesis of MM (68).

Although very intriguing, these reports require few words of explanation. Only in the subset of MM patients, the abnormal immune response to infection may play a role in the pathogenesis of MM (69). However, the infectious agent was not detected or remains unknown in the rest of them.

Considering the reports about the role of an abnormal immune response against infectious agents in the pathogenesis of MGUS and MM, it seems highly probable that the continuous and large-scale interactions between B cells and the gut microbiota could play a role in the pathogenesis of gammopathies. Therefore, it is crucial to detect whether the monoclonal Ig is targeting specifically against some bacteria that colonize the gastrointestinal tract and whether temporal changes in the composition of the gut microbiota could influence the initiation of gammopathies.

## 4 The Interaction Between Gut Microbiota and Other Cells

### 4.1 Epithelial Cells

Recent studies showed that gut epithelium plays a critical role in regulating the host immune system and the luminal microbiota. Intestinal epithelial cells (IECs) include Paneth cells, absorptive epithelial cells, and goblet cells, and their two central roles are to segregate and mediate between the microorganisms colonizing the gut and the immune system. The former function of IECs is possible because of the physical and chemical barriers which prevent the intestinal inflammation that could start because of conflict between these “two armies’’ of cells. The latter function means that IECs can forward signals deriving from the gut microbes and their metabolites and transform that signal for the language “understandable’’ for the immune cells (70). One example of such crosstalk where IECs play a crucial role was seen in mice when segmented filamentous bacteria (SFB) colonized the gastrointestinal tract of germ-free mice attached to the surface of the IECs and induced the production of serum amyloid A (SAA) (52). That, in turn, caused the facilitation of the Th17 differentiation and IL-23 receptor-dependent IL-22 production by innate lymphoid cells 3 (ILC3) (71). On the other hand, IL-17 and IL-22 from Th17 and ILC3 cells induce the production of antimicrobial molecules such as antimicrobial peptides (AMPs) and the regenerating islet-derived 3 (Reg3) family of proteins by epithelial cells, which control the composition of the gut microbiota (72).

What is particularly important from the point of view of this review is to know that IECs drive the IgA class switching in B cells, which are occupying the lamina propria, *via* the production of a proliferation-inducing ligand (APRIL) through toll-like receptor (TLR) signaling (73). The fraction of cells mainly engaged in that process is the M cells, which specialize in the uptake and delivery of antigens derived from the lumen to the antigen-presenting cells (APC) such as dendritic cells (74). Essential for that aim is glycoprotein A (GP2), a transcytotic receptor of M cells responsible for transporting antigens from the lumen to the other side of the wall (75).

Therefore, as was presented, the IECs are responsible for the transition of signals (by TLRs and other receptors and M cells) between the gut microbes and the immune cells that are staying on the two sides of the wall and by secretion of chemokines, cytokines, and hormones they maintain the balance between “both armies.”

### 4.2 Immune Cells

**Figure 1:**
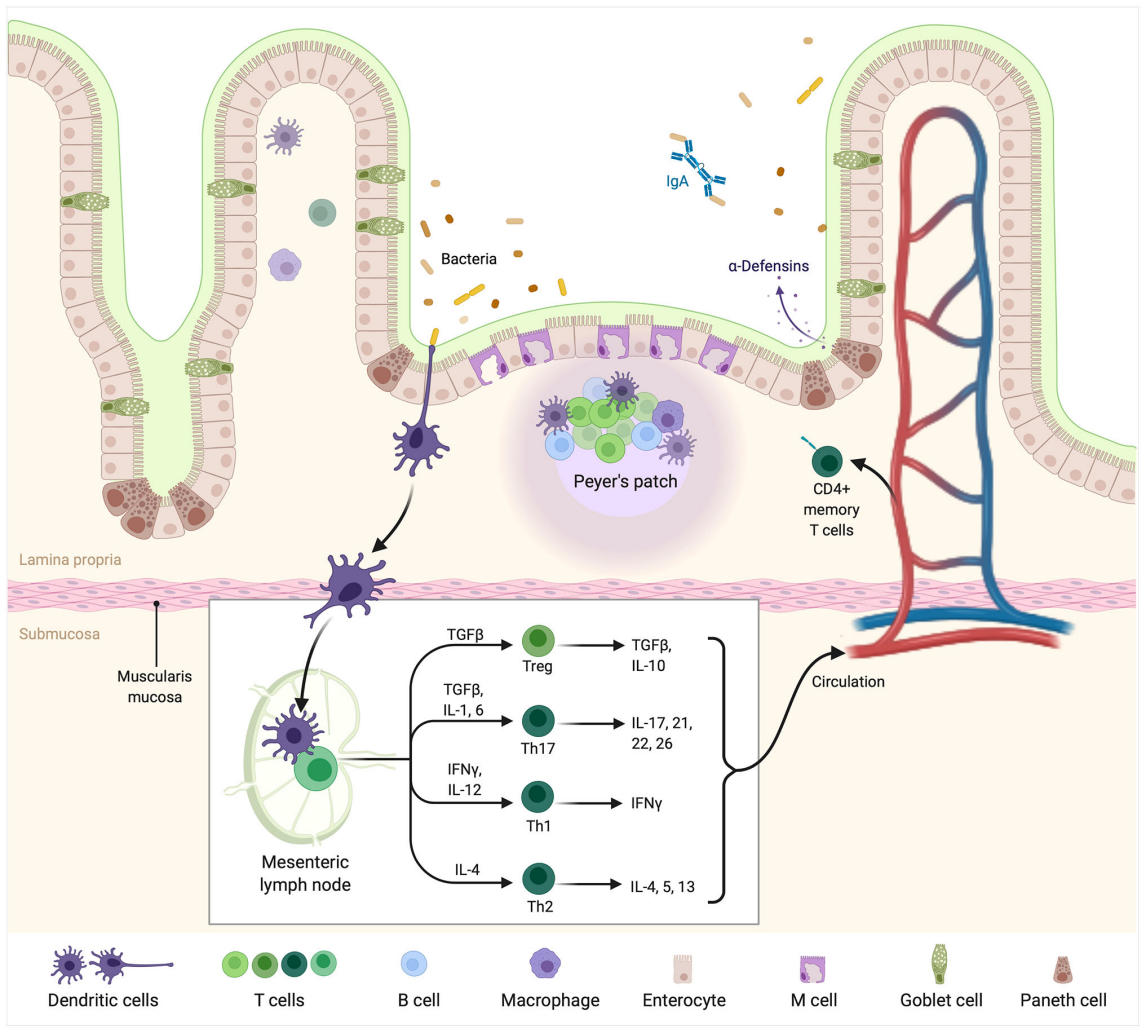
The intestinal immune system. Bacteria currently colonizing the gut are sensed by DCs presenting their antigens in mesenteric lymph nodes or Peyer’s patches. In lymph nodes, DCs are accountable for further differentiation of T cells into Treg, Th17, Th1, and Th2 cells producing pro or antiinflammatory “profile” of cytokines. From the “myeloma point of view” particularly important is the balance between Treg/Th17 cells. The latter is accountable for pro-inflammatory cytokines such as IL-17 production, which are known to facilitate the development of multiple myeloma. Created with BioRender.com.

Immune cells engaged in the crosstalk with the gut microbiota are predominantly seen in the lamina propria. The most common ones are T regulatory cells, NK cells, and invariant T cells. Dendritic cells infiltrate very deeply into villi and closely contact with the IECs (76) (**Figure 1**).

#### 4.2.1 Dendritic Cells (DCs)

The immune cells at the site of the gut epithelium should generate tolerance to the antigens found in the food, but simultaneously they must be ready for immediate response to the emergence of pathogens. DCs are APCs known to be the central players in the immune system together with macrophages. These cells can uptake the antigens from the gut microbes with the mediation of epithelial cells or directly extend their dendrites through the inner mucosal lining to connect with the environment colonized by the microorganisms (77). Through those mechanisms, the DCs eventually shape the composition of the gut microbiota by sampling the microbes and then giving the special orders to activate appropriate response (78). The key role processes occur in the mesenteric lymph nodes where the antigens derived from the lumen are presented to the naïve T cells by the DCs (79). These DCs are characterized by the inability to leave the mesenteric lymph nodes and reach the spleen, thus preventing the organism from inducing a commensal-specific systemic response (80). The specific type of DCs, occupying the lamina propria is characterized by the expression of CD103 on its surface and the production of TGF-β, which causes the differentiation of naïve T cells into CD4+CD25+Foxp3+ T cell of regulatory phenotype (81). This is particularly peculiar given the fact that usually DCs release the inflammatory cytokines and drive the differentiation of Th1 cells. Therefore, researchers hypothesize that the local environment of IECs can stimulate this specific phenotype of DCs. That local environment means, for instance, the thymic stromal lymphopoietin (TSLP) released by the IECs, which was shown in humans to induce the release of the APRIL and BAFF by DCs and in turn supports the class switching of the B cells to IgA (82) or the switching of IgA1 to the IgA2 production which are characterized with protease-resistant phenotype (73). Nevertheless, as will be mentioned further, the epithelial cells are not the only ones to modulate the function of DCs because the other immune cells, like macrophages, can also regulate the function of that population.

#### 4.2.2 Macrophages

Macrophages share some similarities with CD103+ DCs. One of them is the ability to induce the differentiation of the Treg cells (83). However, contrary to the DCs, macrophages’ migration to the mesenteric lymph nodes has not been shown yet, so they probably do not induce oral tolerance (79). One of the existing hypotheses is that macrophages associated with the gut epithelium support the maintenance of Treg cells. Additionally, macrophages can tune the proinflammatory function of DCs by inhibiting their ability to drive Th17 differentiation (83).

Unlike the macrophages residing other than gut tissue, the subtype of macrophages associated with the gut does not possess the CD14 on their surface, responsible for the LPS-induced cell activation and proinflammatory cytokines production (84). Furthermore, these cells produce anti-inflammatory cytokines such as IL-10 and help the DCs maintain the population of Treg to prevent the mucosal auto-inflammation (83). However, although human gut macrophages are known for anti-inflammatory functions, they do not lose their ability to phagocyte and perform defense functions (84). There is also known that within the gut, the population of CD14+ macrophages reside and can produce proinflammatory cytokines such as IL-23 and TNF-α, leading to the further accumulation of similar cells (85).

#### 4.2.3 Neutrophils

Flagellin is the protein of gram-negative bacteria such as *Proteus* or *Escherichia* that stimulates the TLR5/MyD88 signaling in IECs. This pathway promotes the production of IL-8 by IECs, which causes the recruitment of neutrophils to the lamina propria (86).

Neutrophils are known to promote or inhibit the growth of the tumor (87). Moreover, they can switch from promoting to the inhibiting mode and stop the tumor’s progression (88). On the other hand, the interactions between neutrophils and the gut microbiota were shown to impact the tumor’s growth rate. An example of that is a mouse model of serrated polyps, a premalignant lesion of the colon. Throughout the intestine, the endothelial growth factor receptor ligand is produced, but only the cecum is the site where that molecule promotes the development of polyps. That is because the growth of polyps requires the specific gut microbiota composition in the cecal mucosa. Furthermore, it was shown that administration of antibiotics or depletion of neutrophils resulted in inhibition of serrated polyps’ development, suggesting the crucial role of bacteria and neutrophils in that process (89).

#### 4.2.4 T Cells

We limit the interplay between T cells and the gut microbiota to the role of the Th17/Treg cells balance because of their great importance in switching the mode of the immune system from pro to anti-inflammatory and vice versa. Such imbalance was reported to play a role in chronic inflammations (90), allergic diseases (91), cancers, and autoimmune diseases (92, 93). Germ-free mice were shown to have decreased number of both populations of cells (94), but it has also been shown that specific metabolites such as ATP and short-chain fatty acids (SCFA) can induce differentiation of Th17 and Treg cells, respectively (95, 96). SCFA are the bacterial products produced from the dietary fiber by the anaerobic gut microbiota (97).

#### 4.2.5 B Cells and Plasmacytes

The gut microbiota is known for its impact on the development, differentiation, activation, and function of the B cells. Regarding the development of human innate-like B cells and marginal zone B cells, the gut-associated lymphoid tissue (GALT) may be the site of a growing repertoire of B cells (98). Interestingly, a subset of human immature B cells, known as transitional 2 (T2) B cells from the bone marrow, tend to reside in the intestine for their activation. The maturation process relies on eliminating self-reactive B cells from the developing repertoire. Failure in that process is seen in the systemic lupus erythematosus (SLE), which suggests that this site constitutes a checkpoint against autoimmunity (99).

The intestinal microbiota may influence the B cells through the direct and indirect modes. The former depends on B cells’ direct activation *via* BCR recognition of the carbohydrates and proteins produced by the gut microbes, which act as antigens (100). That is T-dependent B-cell activation, but B cells can be activated T cell-independently. That is because they have TLRs on their surface, which are extremely important for their survival and function (101). For example, Oh et al. showed that mice lacking the TLR5 could not develop immunity against seasonal influenza vaccination because of the inability to sense the gut microbiota (102). Similarly, the metabolites of the bacteria can also activate the B cells. For instance, SCFAs may affect B-cell metabolism and facilitate the differentiation of B-cell, hence promoting immunoglobulin promotion (103).

**Figure 2:**
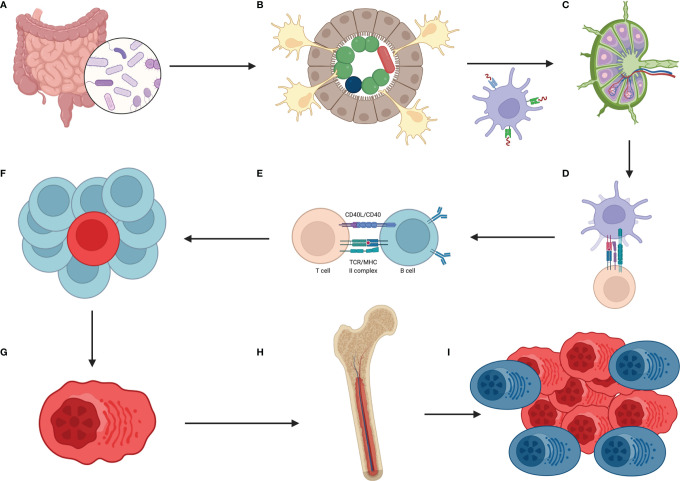
How the hypothetical pathway from dysbiosis to multiple myeloma looks? The sequence of events is as follows **(A)** lack of balanced gut microbiota which means overgrowth of selected species of bacteria **(B)** these bacteria are accountable for constant, oligo- or even monoclonal stimulation of DCs (with help of IECs) which migrate to mesenteric lymph nodes and/or Peyer’s patches **(C)** there, B cells, T cells and mentioned DCs meet each other **(D)** DCs are presenting this oligo-, monoclonal antigens to T cells **(E)** which then are responsible for selection of B cells that are going to have required features to combat the antigen **(F)** because of continuous stimulation in the gut the process of B cell selection is intense and these cells undergo numerous rounds of proliferation which are preceded by SHM and class switching, associated with DNA changes **(G)** one initial B cell with driver mutation emerges, transforms to plasma cell that produces oligo-, monoclonal antibodies against the antigen, and proliferates **(H)** then plasma cells migrate to bone marrow which is the site of constant immunoglobulins production **(I)** when mutated plasma cells acquire additional mutations and are surrounded by favorable milieu then initial state of MGUS changes into SMM, MM and eventually to PCL. Created with BioRender.com.

Although some papers showed in recent years that a repertoire of B cells could develop within GALT with the help of the gut microbiota, there are still many gaps in that field. However, that potentially shed light on the possibility that the composition of the gut microbiota could shape the repertoire of B cells and that dysbiosis could be potentially accountable for the chronic antigenic stimulation of B cells and subsequent genesis of MGUS and MM (**Figure 2**).

## 5 Possible Mechanistic Pathway of Myeloma Cell Initiation in the Context of Microbiota

It is well known that the IgA is produced in the intestine by B cells, but little is known about the production of other subtypes of immunoglobulins. However, there is already evidence that the gut microbiota may induce the TLR4-dependent production of IgG and that these antibodies are efficient in fighting against systemic infection (104). Furthermore, some other studies show that the SCFAs regulate the production of immunoglobulins in different ways. For example, it was shown that after the administration of cholera toxin, the SCFAs facilitate the production of BAFF and retinoic acid (RA) by DCs to upregulate the synthesis of IgG and IgA (105). Given that, it is reasonable to think that SCFA may play an active role in regulating the production of immunoglobulins.

Considering what was said before that the gut microbiota could potentially drive the repertoire of BCR, it seems probable that dysbiosis could affect that process. MGUS starts when the monoclonal globulin starts to be detectable and when there are less than 10% of clonal plasma cells within the bone marrow. Nevertheless, the first mutated cell is probably created a long time before the diagnosis of MGUS or MM. Therefore, we speculate that this first step towards entirely symptomatic MM could start within the gastrointestinal wall. Given that microbes, their antigens, and metabolites are recognized by B cells, activate them, and provoke proliferation, it seems reasonable to think that lack of balanced gut microbiota with overgrowth of sparse species of bacteria and then chronic antigen stimulation could lead to fully symptomatic MM. Thus, a mechanistic vision of gut microbiota-dependent myeloma formation could be like we deliberate below.

Because of acquired or resulting from genetic predispositions dysbiosis, there is an overgrowth of selected microorganisms in the gut. Sometimes, even subtler changes such as overgrowth of one bacteria species or even the presence of one antigen that is constantly produced within the gut by microbes could constantly stimulate the immune system of the GALT. Then DCs occupying this area are continuously activated with the help of IECs in that process. DCs, after first contact with antigen, are migrating to mesenteric lymph nodes, which is the place of the “crime,” where B cells are stimulated by a minimal number of antigens presented by DCs. That stimulation leads to numerous rounds of proliferation done by B cells, during which they are accumulating mutations during the processes of SHM and class switching. Eventually, the first mutated cell emerges, but that does not necessarily mean that progression of MM is initiated here. Changed plasma cells after rounds of proliferation reach the bone marrow, where they will produce immunoglobulins. As said before, the additional events must occur within the bone marrow to facilitate the progression from MGUS, through SMM, to fully symptomatic MM, and eventually to PCL. That progression is supported by the proinflammatory cytokines, which are stimulating osteoclasts to destroy the bones to “make space’’ for quicker and quicker proliferating myeloma cells (106). We postulate that this is another process in which the gut microbiota could play the role since papers show that the lack of balanced gut microbiota results in a more proinflammatory state of the immune system, and for instance, differentiation of T cells within the gut is skewed towards Th17 cells. Moreover, the work of Jian et al. showed that “crosstalk on distance” of myeloma cells and the gut microbes is possible and that these two groups of cells cooperate and support the growth mutually (107).

Considering that, it is worth asking whether there are any changes in the gut microbiota between different stages of the disease from MGUS to PCL? An initial, small study done by Pepeljugoski et al. proves that such changes occur (108) and that progression in the disease is associated with developing dysbiosis. Additionally, it would be very interesting to check if dysbiosis or even subtle changes in the gut microbiota composition could be a risk factor for MGUS. Perhaps, at least some of the cases of MGUS/MM are producing a monoclonal protein targeting antigens deriving from the gut. That hypothesis will be discussed further, but to show that dysbiosis is a critical player in the progression of the disease, a correlation study that will link the gut microbiota composition with the immune-related gene expression profile is needed. Our group has initiated a study on newly diagnosed MGUS, SMM, and MM patients recently, in which we are going to search whether there is such correlation and how it is changing with time and applied treatment.

## 6 The Effects of Gut Microbiota Composition on Treatment Results in MM

Lack of balanced gut microbiota can lead to the lack of “training’’ given to the immune system by microbes. That, in turn, can lead to the inhibition of the active immune system that can combat new cancer cells created every day. Therefore, along with changing the paradigm to an immune-dependent approach, the gut microbiota role in the effectiveness of immunotherapy or cellular therapy should be revised. For instance, it is probable that by restoring the balanced gut microbiota, the results of mentioned therapies could be enhanced. Such a prove we can learn from immune checkpoint inhibitors and cancer treatment (109). However, this could also lead to more pronounced adverse events (110).

Cyclophosphamide was shown with very low efficacy when mice were injected with tumor cells and then treated with antibiotics to achieve a germ-free microenvironment. Thus, a lack of balanced gut microbiota causes low sensitivity of tumor cells for cyclophosphamide (111).

Autologous stem cell transplantation (ASCT) is currently a standard of care for patients in good condition. Until the engraftment, the patients are in critical pancytopenia and prone to opportunistic infections and therefore very usually treated with antimicrobials. However, before that, patients receive conditioning therapy that influences the composition of the gut microbiota and has a gross impact on intestinal epithelium. That altogether leads to the dysbiosis and monodominance of microbes such as Enterobacteriaceae (112). Researchers have also shown that butyrate, one of the SCFAs produced by the *Eubacterium halii* and *Faecalibacterium prausnitzii*, is associated with minimal residual disease negativity after the induction therapy for MM (113).

Regarding the proteasome inhibitors (PIs), which are commonly used to treat MM, it is worth noting that their adverse event is diarrhea. Unfortunately, the pathophysiology of gastrointestinal toxicity of PIs is poorly understood. However, as it is known that the SCFAs and PIs regulate the NF-kB pathway, the gut microbiota probably influences the risk of adverse events after PIs (114).

## 7 Novel Approaches in Methods Exploring the Role of Gut Microbiota in MGUS and MM Development

Calcinotto et al. published a study on mice where they showed that one specific species of the microorganisms colonizing the gut, namely *Prevotella heparinolytica* induced differentiation of Th17 cells. They then migrated to the bone marrow of Vk*MYC mice (which are the transgenic mice that develop disease mimicking MM) and favored the progression of MM. That agrees with the notion that SMM patients with a higher level of IL-17 in the bone marrow have faster progression of the disease (115).

Moreover, the relationships between myeloma cells and the gut microbes should be elucidated by identifying the gut microbiota composition that predicts a higher probability of MGUS development. It would be essential to know the specificity of the monoclonal protein produced by the mutated clone and correlate the results with the gut microbiota. A hypothesis is that pathogenic species colonizing (even temporally) the gut or state of dysbiosis when particular species of bacteria overgrowth could be responsible for the chronic antigenic stimulation and development of at least part of MGUS and MM cases.

Additionally, it would be interesting to see whether the gut microbiota composition influences the cytokines produced by the leukocytes in the blood. For example, maybe the state of dysbiosis provokes the production of proinflammatory cytokines by leukocytes and, therefore, indirectly promotes the progression of MM or MGUS.

## 8 New Potential Targets of Treatments in Multiple Myeloma

As it was said in the previous paragraph, it is worth checking whether the treatments targeting IL-17 or IL-17R could work by lowering the risk of progression of the MGUS/SMM/MM. Such drugs are already registered by the FDA (anti-IL-17A antibodies), making it even easier to conduct such a study (116).

Preclinical studies suggest that some species of bacteria colonizing the gut promote the progression of MGUS or MM. Papers are mounting about the role of the gut microbiota in the pathogenesis of many diseases, and great hope is seen in the procedure of fecal microbiota transplantation to restore the balanced gut microbiota. Maybe such a procedure or probiotics/prebiotics could lower the risk of progression of MGUS to more advanced stages of the disease. Currently, the patients with MGUS or SMM are offered with watchful waiting strategy since the risk of progression, especially in the case of MGUS, is particularly low. Perhaps in the future, the gut microbiota composition of these patients is going to be known in detail. The patients with an exceptionally high risk of progression could be treated with prebiotics/probiotics or even with fecal microbiota transplantation (FMT) to diminish the risk of progression completely.

Jian et al. showed recently that the bacterial diversity of the gut microbiota of newly diagnosed MM patients is significantly increased with enrichment of nitrogen-recycling bacteria such as *Klebsiella* and *Streptococcus*. The researchers assume this is because of the progression of MM associated with the excessive accumulation of urea. Then the urea reaches the intestinal wall and selects nitrogen-recycling bacteria for overgrowth. In turn, microbes can produce L-glutamine, which is then delivered to the host and promotes the proliferation of myeloma cells since they cannot produce it on their own. Thus, the authors propose that the gut microbiota alterations, namely reduction of *Klebsiella* and *Streptococcus* populations, which are also present in the normal microflora, could lower the risk of progression of MM (107). Furthermore, MM patients are also prone to infections, for instance, pneumonia with a common etiology of *Klebsiella* or *Streptococcus* (117). Therefore, the reduction of these populations could additionally mitigate the risk of infections during the disease. Additionally, it was also found that SCFA-producing bacteria were depleted in MM, and the addition of such bacteria in mice resulted in the mitigation of tumor progression (107).

## 9 Conclusions

To sum up, it seems highly probable that there is a role of the gut microbiota in the pathogenesis and treatment of MM. With ever-growing numbers of papers published in that field, the hope for an entirely new type of prophylaxis of progression of MGUS or treatment of MM is growing in parallel. Our previous work shows the remarkable efficacy of FMT in preventing colonization of a single, in that case, antibiotic-resistant bacteria (118). Also, in the model of graft-versus-host disease, we have shown that it is possible to stop the inflammatory process in the gut by FMT, shedding new light on the immunomodulatory effect of the gut microbiota (119, 120). Given that single species of bacteria, *Klebsiella* and *Streptococcus* were shown to play a role in the progression of MM, it seems that further studies on gut microbiota in the treatment of MM are warranted. Additionally, these bacteria are often responsible for infections in that population of patients. Therefore, the possible efficacy of FMT in the elimination of these “microbial partners in crime’’ would be multidirectional.

## Author Contributions

MJ and JB wrote the paper, GWB reviewed the paper. All authors contributed to the article and approved the submitted version.

## Conflict of Interest

JB and GWB are the founders of the fecal microbiota bank and laboratory named the Human Biome Institute.

The remaining author declares that the research was conducted in the absence of any commercial or financial relationships that could be construed as a potential conflict of interest.

## Publisher’s Note

All claims expressed in this article are solely those of the authors and do not necessarily represent those of their affiliated organizations, or those of the publisher, the editors and the reviewers. Any product that may be evaluated in this article, or claim that may be made by its manufacturer, is not guaranteed or endorsed by the publisher.
